# Regulating the Optoelectronic Properties of Nickel Dithiolene by the Substituents: A Theoretical Study

**DOI:** 10.3390/ma11112192

**Published:** 2018-11-06

**Authors:** Lili Sun, Siwei Shu, Yi Zhou, Sen Hou, Yan Liu, Zhuofeng Ke

**Affiliations:** 1 School of Materials Science and Engineering, PCFM Lab, Sun Yat-sen University, Guangzhou 510275, China; sllmnls@126.com (L.S.); zhouy348@mail2.sysu.edu.cn (Y.Z.); 2 School of Chemical Engineering and Light Industry, Guangdong University of Technology, Guangzhou 510006, China; shusiwei1994@163.com (S.S.); yanliu@gdut.edu.cn (Y.L.); 3 School of Environment, Guangzhou Key Laboratory of Environmental Exposure and Health, Guangdong Key Laboratory of Environmental Pollution and Health, Jinan University, Guangzhou 510632, China

**Keywords:** nickel dithiolene, substituents, optoelectronic properties, DFT

## Abstract

Dithiolene-based complexes show great potential to be applied as materials for organic optoelectronic devices. In this study, we theoretically designed a series of complexes based on nickel dithiolene and its substituted derivatives, the optoelectronic properties of which were comparatively studied by density functional theory (DFT)/time-dependent density functional theory (TD-DFT). The results show that the charge injection property of nickel dithiolene complexes can be significantly improved with introduction of electron-withdrawing groups. The charge transportation property of nickel dithiolene depends on the conjugation degree of the system. The energy gaps between highest occupied molecular orbital (HOMO) and lowest unoccupied molecular orbital (LUMO) are determined by the substituents, which makes the maximum absorption wavelength red-shift from the visible to the near-infrared (NIR) region. The electron density difference graph shows that the electron transition from the ground state to the first excited state is assigned to π-π* transition mainly from HOMO to LUMO. The regularity of substituent effect revealed by us in this study will shed light on the application of nickel dithiolenes as potential optoelectronic materials.

## 1. Introduction

Neutral nickel bis-dithiolene complexes have recently attracted considerable interest as new potential materials in a wide-span range of optoelectronics devices such as ambipolar channel field effect transistors (FETs), thin film solar cells, second or third order non-linear optical devices, near-infrared (NIR) liquid crystal devices, and so on [1,2,3,4,5,6]. Recently, dithiolenes complexes have re-emerged as hydrogen evolving catalysts to generate H_2_ from H^+^ for artificial photosynthesis [7,8,9]. Nickel dithiolenes can be applied as materials for these optoelectronics devices due to their thermal and photochemical stability [10,11], widely tunable and reversible redox properties [12,13], their square planar structures which allow close packing in the solid state [2], film formation feasibility, high electron mobility, and their intense absorption in the NIR region [14]. Their potential application for the n-channel semiconductor materials is of vital importance since the semiconducting materials for the n-channel semiconductor are scarce [15,16]. Besides this, nickel dithiolenes have been applied as sensitizers in dye-sensitized solar cells (DSSCs), where they are supposed to capture light and their electrons are easily injected into the semi-conducting materials of the DSSCs [10]. Herein we explore the feasibility of applying nickel dithiolenes as n-channel semiconducting materials and DSSCs materials.

Although a large number of molecular and polymeric species have been experimentally and theoretically found as good materials for p-type semiconducting materials, only a limited number of compounds belongs to the n-type materials [16]. Nickel dithiolenes have been experimentally reported to be of very high electron mobility. Thus, they have been suggested as n-type material since 1994 [17]. Theoretical study at the molecular level is useful not only for a better understanding of relationship between chemical structure and properties, but also it provides us a tool for designing new n-type semiconducting materials. Unfortunately, theoretical studies for n-type semiconducting material design are scarce until now.

Owing to their ability to absorb NIR photons, nickel dithiolene complexes have been considered as candidates for NIR sensitizers in dye-sensitized solar cells in recent years [10]. Nickel dithiolene complexes show strong crystal field and long-lived excited states [18]. The charge injection into the semiconductor layer is considered as the limiting factor for the life of excited states [19]. Nevertheless, the efficiency of DSSCs device based on nickel dithiolenes is still low [2,19,20].

The key to apply nickel dithiolenes as the materials for n-type semiconductor and NIR sensitizers of DSSCs lies in their electron configuration and electronic transition properties. The electron configuration and transition of nickel dithiolenes can be regulated by altering the substituents. For example, Ni(S_2_C_2_R_2_)_2_ (R = –CF_3_, –CN, –H, –Ph, –CH_3_) can be reduced to [Ni(S_2_C_2_R_2_)_2_]^−^, and the redox potentials depend on the substituents [13]. The substituents also affect the energy and intensity of low-lying electronic transition of nickel dithiolene [12]. For example, Fan et al. found that the energies of the highest occupied molecular orbital (HOMO) and the lowest unoccupied molecular orbital (LUMO) of Ni[S_2_C_2_(CF_3_)_2_]_2_ and Ni[S_2_C_2_(CN)_2_]_2_ are much lower than those of Ni(S_2_C_2_H_2_)_2_ [21]. Moreover, the substituents affect electron orbital composition of nickel dithiolene. For example, the substituent −CN as electron acceptor can considerably reduce electron density of S atoms in Ni[S_2_C_2_(CN)_2_]_2_ [22].

In this study, we theoretically designed the materials for n-type semiconductor and NIR sensitizer for DSSCs based on nickel dithiolene. We studied the effect of substituents on the optoelectronic properties of nickel dithiolene. The substituents considered in this study are –NO_2_, –CN, –CF_3_, –F, –H, –CH_3_, –C_2_H_5_, –CH=CH_2_, –C≡CH, –OH, –NH_2_, most of which are commonly used electron-withdrawing or electron-donating groups. The geometrical structures, the frontier molecular orbitals, the ionization potentials, the electron affinities, the reorganization energies, the electronic absorption spectra and the light harvesting efficiency of nickel dithiolenes are comparatively studied by density functional theory (DFT) methods.

## 2. Computational Methods

The geometrical structure and the frontier molecular orbital of nickel dithiolenes were calculated by the GAUSSIAN 09 software package program [23]. The calculations were carried out by a hybrid density functional, namely, Becke’s three-parameter nonlocal hybrid exchange-correlation functional B3LYP [24]. The outer valence orbital of Ni was based on LANL2DZ basis set [25], and the two outermost *p* functions were replaced by a (41) split of optimized *p* function for Ni atom [26]. The f polarization function was also added to Ni atom in order to improve the accuracy [27]. The basis set for other atoms was 6-311 + G*. All the optimum structures were confirmed by the hessians. The second derivatives provided harmonic vibrational frequencies to verify the nature of the stationary points, which were not additionally corrected in this paper.

The ionization potential (IP) includes the vertical ionization potential (IP(v)) and the adiabatic ionization potential (IP(a)). IP(v) and IP(a) are defined as

(1) $$\mathrm{IP}\left(v\right)=E_{+}^{*}-E_{0}\;\mathrm{and}\;\mathrm{IP}\left(a\right)=E_{+}-E_{0}$$

where $E_{0}$ represents the energy of neutral state in the optimized neutral geometry, $E_{+}^{*}$ represents the total energy of cationic state in the optimized neutral geometry, $E_{+}$ represents the total energy of cationic state in the optimized cationic geometry. Generally, IP(v) is larger than IP(a) because $E_{+}^{*}$ is higher than $E_{+}$.

The electron affinity (EA) is composed of the vertical electron affinity (EA(v)) and the adiabatic electron affinity (EA(a)). EA(v) and EA(a) can be expressed as

(2) $$\mathrm{EA}\left(v\right)=E_{0}-E_{-}^{*}\;\mathrm{and}\;\mathrm{EA}\left(a\right)=E_{0}-E_{-}$$

where $E_{-}^{*}$ represents the total energy of anionic state in the optimized neutral geometry, $E_{-}$ represents the total energy of anionic state in the optimized anionic geometry. Generally, EA(a) in the study is larger than EA(v) because $E_{-}^{*}$ is larger than $E_{+}$.

The reorganization energy for electron transfer ($\lambda_{\mathrm{electron}}$) is defined as

(3) $$\lambda_{\mathrm{electron}}=\lambda_{0}+\lambda_{-}=\left(E_{0}^{*}-E_{0}\right)+\left(E_{-}^{*}-E_{-}\right).$$

The reorganization energy for the hole transfer $\lambda_{hole}$ is defined as

(4) $$\lambda_{hole}=\lambda_{0}+\lambda_{+}=\left(E_{0}^{*}-E_{0}\right)+\left(E_{+}^{*}-E_{+}\right)$$

where $E_{0}^{*}$ represents the energy of neutral species with optimized anion/cationic geometry in Equations (3) and (4).

The electron absorption spectra of nickel dithiolene and its substituted derivatives were predicted in gas phase using time-dependent density functional theory (TD-DFT) based on the optimized ground-state structures. The electronic absorption spectra of Ni(S_2_C_2_H_2_)_2_ in solvent was simulated by using the polarized continuum model (PCM) [28] in order to compare the calculated maximum absorption wavelength with the experimental data.

The light harvesting efficiency (LHE) prominently affects the Incident-photon-to-electron Conversion Efficiency (IPCE), which can be expressed as [29]

(5) $$\mathrm{LHE}=1-10^{-f}$$

where *f* represents the oscillator strength of the dye associated to the maximum absorption wavelength (at first order, LHE is proportional to f).

## 3. Results and Discussion

### 3.1. Verification of Computational Method

To verify the reliability of DFT methods, we compared the optimized bond lengths and bond angles of Ni(S_2_C_2_H_2_)_2_, Ni[S_2_C_2_(CH_3_)_2_]_2_, and Ni[S_2_C_2_(CN)_2_]_2_ with the available experimental data. Geometry optimization were performed by four kinds of functionals including M06, M06L, PBEPBE, and B3LYP. For all these four functionals, the bond lengths and bond angle of C–S, Ni–S, C–C, and S–Ni–S in Ni(S_2_C_2_H_2_)_2_, Ni(S_2_C_2_[(CH_3_)_2_]_2_, and Ni[S_2_C_2_(CN)_2_]_2_ agreed well with the available experimental data, in which the error of bond length was less than 0.01–0.02 Å and the error of bond angle was less than 1°. (Table S1) TD-DFT calculation was performed to compare the computed maximum absorption wavelength of Ni(S_2_C_2_H_2_)_2_ in hexane with the experimental data. The predicted maximum absorption wavelength by B3LYP (714.15 nm) was much better than that predicted by M06, M06L, and PBEPBE (Table S2) and it agreed well with the experimental value (719 nm). Therefore, we chose the B3LYP functional to perform DFT calculation in this work.

### 3.2. Geometry and Frontier Molecular Orbitals

**Figure 1:**
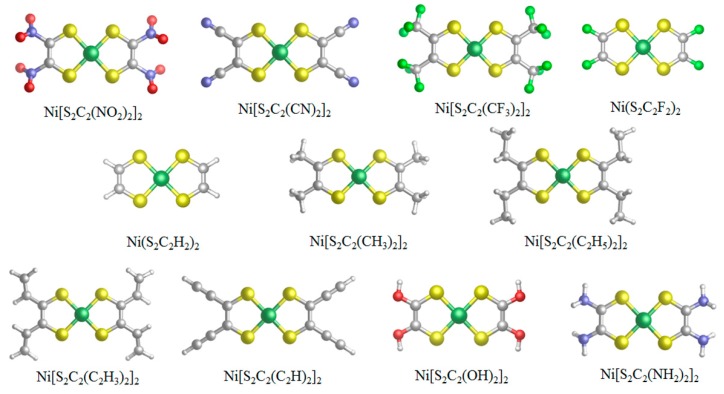
Geometries of Ni(S_2_C_2_R_2_)_2_ (R = –NO_2_, –CN, –CF_3_, –F, –H, –CH_3_, –C_2_H_5_, –CH=CH_2_, –C≡CH, –OH, –NH_2_) optimized by Gaussian 09 software. Ni atom is in dark green; O atom is in red; N atom is in purple; S atom is in yellow; C atom is in gray; H atom is in white; F atom is in light green.

We investigated the effect of substituents on the optoelectronic property of Ni(S_2_C_2_H_2_)_2_. Both the electron-withdrawing substituents (–NO_2_, –CN, –CF_3_, –F) and the electron-donating substituents (–CH_3_, –C_2_H_5_, –OH, –NH_2_) were considered. The electrophilic properties of alkene and alkyne substituents (–CH=CH_2_, –C≡CH) are close to that of –H. They are conjugated with the dithiolene ring (Figure 1). They extend the length of the conjugated system, which is helpful to improve the transportation ability of charge carrier [16]. Therefore, the effects of –CH=CH_2_ and –C≡CH on the optoelectronic property of Ni(S_2_C_2_H_2_)_2_ were also studied.

The geometry structures of nickel dithiolenes were optimized by Gaussian 09 software. The two chelated pentatomic rings of Ni(S_2_C_2_H_2_)_2_ were coplanar and highly symmetric. The introduction of substituents did not make the two chelated pentatomic rings out of plane. Especially, the linear and unsaturated groups (–C≡CH and –C≡N) were conjugated with two chelated pentatomic rings (Figure 1). In the optimized cationic state or the anionic state, the two chelated pentatomic rings of nickel dithiolenes were still approximately coplanar, which ranges from 179° to 180°. We expect that this highly coplanar structure will provide their solid state with more packed crystallization structure and subsequently will improve the charge mobility in the solid state.

The HOMO and LUMO of substituted nickel dithiolenes are mainly composed of dithiolene and substituents orbital character. The atom contour of substituents is approximately parallel with that of dithiolene. Therefore, there should be orbital interactions between dithiolene and substituents. We optimized the frontier molecular orbitals of nickel dithiolenes by Gaussian 09 software. The unsaturated substituents −NO_2_, –CN, –CH=CH_2_, and –C≡CH conjugate with two dithiolene ligands. The p orbitals of lone pairs of F, O, and N atoms conjugate with the dithiolene ligand. The *σ* orbitals of –CF_3_, –CH_3_, and –C_2_H_5_ contribute to the composition of HOMO and LUMO of nickel dithiolene (Table S3). Hence, it is very likely that the substituents affect the energy of the frontier orbitals, electron transfer, and electron transition of nickel dithiolenes.

We found that the energy levels of HOMO/LUMO of Ni(S_2_C_2_H_2_)_2_ were significantly affected by both the electron-withdrawing substituents and the electron-donating substituents. The energy levels of HOMO/LUMO of Ni(S_2_C_2_H_2_)_2_ were higher than those of nickel dithiolenes with electron-withdrawing groups (such as –NO_2_, –CN, –CF_3_, –F), and lower than those of nickel dithiolenes with electron-donating groups such as (–CH_3_, –C_2_H_5_, –OH, –NH_2_). The result agrees with a previous report that electron-withdrawing/donating groups tend to lower/raise the energy levels of the conjugated systems, respectively [30]. With the introduction of substituents, with the exception of the trifluoromethyl derivative, the energy gaps between HOMO and LUMO became lower than Ni(S_2_C_2_H_2_)_2_ (Table 1). Therefore, it can be expected that the electron transition from the HOMO to the LUMO will be easier for the substituted nickel dithiolenes than Ni(S_2_C_2_H_2_)_2_.

The HOMO/LUMO plays an important role in evaluating the applicability of organic semiconductors. Either the HOMO energy level of p-type semiconducting material or the LUMO energy level of n-type semiconducting material should be close to the work function (WF) of the electrodes in a vacuum in order to improve their charge transporting ability. In this study we took the commonly used Au electrode for comparison. The WF of Au electrode is ~5.1 eV. As listed in Table 1, the LUMO levels of nickel dithiolenes with substituents (–F, –H, –CH=CH_2_, –C≡CH) are in the range of −4.5 eV to −5.2 eV, which are close to the WF of the Au electrode (~5.1 eV). This feature provides nickel dithiolenes with a bright prospect to be used as n-channel semi-conducting materials.

### 3.3. Ionization Potentials, Electron Affinities, and Reorganization Energy

Ionization potential is qualitatively defined as the amount of energy required to remove the most loosely bound electron, the valence electron, of an isolated gaseous atom or molecule to form a cation. Generally, the first ionization potential approximates WF providing some useful information about the electron emitting behavior of the studied structures. For organic semiconducting materials, the lower of the *IP* value, the easier it is to inject the hole to the hole-transporting layer. We found the substituent made a great contribution to the *IP* values. Both the *IP(v)* values and *IP(a)* values of the substituted nickel dithiolenes were Ni[S_2_C_2_(NO_2_)_2_]_2_ > Ni[S_2_C_2_(CN)_2_]_2_ > Ni[S_2_C_2_(CF_3_)_2_]_2_ > Ni(S_2_C_2_F_2_)_2_ > Ni(S_2_C_2_H_2_)_2_ > Ni[S_2_C_2_(CH_3_)_2_]_2_ > Ni[S_2_C_2_(C_2_H_5_)_2_]_2_ > Ni[S_2_C_2_(OH)_2_]_2_ > Ni[S_2_C_2_(NH_2_)_2_] (Table 2). The result indicates that the stronger the substituents’ electron-withdrawing ability, the higher the *IP* value is. Accordingly, it is more difficult to remove the electron from the HOMO with the increase of the substituents’ electron-withdrawing ability. Ni[S_2_C_2_(NH_2_)_2_]_2_ had the lowest *IP(v)* value and the lowest *IP(v)* among the studied complexes. Unfortunately, the WF of indium-tin oxide (ITO), the commonly used hole injection layer material, is ~4.9 eV [31]. It is lower than the *IP* values of all the studied complexes. Therefore, the *IP* values of Ni(S_2_C_2_R_2_)_2_ are too high for these molecules to be used as the hole injection materials.

EA of an atom or molecule is defined as the amount of energy released or spent when an electron is added to a neutral atom or molecule in the gaseous state to form a negative ion. The higher the EA value, the easier it is to inject electrons from the cathode. We found the electron-withdrawing ability of substituents significantly affected the EA values of nickel dithiolenes. The EA values of the substituted nickel dithiolenes were Ni[S_2_C_2_(NO_2_)_2_]_2_ > Ni[S_2_C_2_(CN)_2_]_2_ > Ni[S_2_C_2_(CF_3_)_2_]_2_ > Ni(S_2_C_2_F_2_)_2_ > Ni(S_2_C_2_H_2_)_2_ > Ni[S_2_C_2_(CH_3_)_2_]_2_ > Ni[S_2_C_2_(C_2_H_5_)_2_]_2_ > Ni[S_2_C_2_(OH)_2_]_2_ > Ni[S_2_C_2_(NH_2_)_2_]_2_, which was in the same order with the IP values (Table 2). Usually, the substituents with stronger electron-withdrawing ability has higher EA values and, in this case, it is easier to inject electron into the LUMO of nickel dithiolene. Moreover, this trend of *EA* accords with the redox potential of nickel dithiolenes from the experimental measurements. The redox potential of Ni[S_2_C_2_(CN)_2_]_2_, Ni[S_2_C_2_(CF_3_)_2_]_2_, Ni(S_2_C_2_H_2_)_2_, Ni[S_2_C_2_(CH_3_)_2_]_2_, and Ni[S_2_C_2_(C_2_H_5_)_2_]_2_ are 1.22 V, 0.92 V, 0.12 V, −0.107 V, and −0.119V, respectively [32]. The electron affinity of commonly used electron injection layer material tris(8-hydroxyquinolate) aluminum (Alq_3_) is between 0.5 eV and 1.5 eV [33]. The calculated EA*(a)* values of nickel dithiolenes were between 4.97 eV and 2.13 eV. The electron affinities of neutral nickel dithiolenes are much higher than those of tris(8-hydroxyquinolate) aluminum. Thus, nickel dithiolenes should be good electron injection layer materials.

In order to evaluate the electron and hole mobilities of a molecule, its reorganization energies for holes and electrons should be considered. The reorganization energies are defined as the sum of geometrical relaxation energies when the species goes from the neutral state geometry to a charged state geometry, and vice versa. Accordingly, a molecule has high charge mobility if it has low reorganization energies. Based on the calculation, we concluded that it would be easier for nickel dithiolenes to transport electron than to transport hole as *λ*_electron_ is smaller than *λ*_hole_ for nearly all the studied complexes (Table 2). The reorganization energy did not correlate with electron-donating or electron-withdrawing ability of substituents. In addition, the reorganization energies were smaller for the unsaturated substituents with π orbital. For example, Ni[S_2_C_2_(NO_2_)_2_]_2_, Ni[S_2_C_2_(CN)_2_]_2_, Ni[S_2_C_2_(CH=CH_2_)_2_]_2_, and Ni[S_2_C_2_(C≡CH)_2_]_2_ had smaller reorganization energies than the rest. Their *λ*_electron_ ranged from 0.102 eV to 0.209 eV and *λ*_hole_ ranged from 0.177 eV and 0.211 eV. These unsaturated groups (–NO_2_, –CN, –CH=CH_2_, –C≡CH) are conjugated with the planar dithiolenes so it becomes easier for the transporting of an electron or a hole. It is worth to note that the *λ*_hole_ and *λ*_electron_ of substituted nickel dithiolenes with conjugated groups are lower than those of tris(8-hydroxyquinolate) aluminum (Alq_3_), the commonly used electron transfer material (*λ*_electron_ = 0.276 eV, *λ*_hole_ = 0.242 eV) [34]. Therefore, they are possible good electron transportation materials.

### 3.4. Electron Transition from the Ground State to the First Low-Lying Excited State

TD-DFT calculations were performed for nickel dithiolene and its substituted complexes using B3LYP basis functional. The maximum absorption wavelengths of substituted nickel dithiolenes in gas phase ranged from 670.76 nm to 1022.86 nm, which were in the visible or NIR region (Table 3). The maximum absorption wavelengths of all the substituted nickel dithiolenes were featured with a red-shift, a decrease in the excitation energies and an increase in the oscillator strengths in comparison to Ni(S_2_C_2_H_2_)_2_. Particularly the maximum absorption wavelengths of Ni[S_2_C_2_(OH)_2_]_2_ and Ni[S_2_C_2_(NH_2_)_2_]_2_ exhibited the largest red-shift, which were 967.89 nm and 1022.86 nm, respectively (Table 3).

Owing to the strong NIR absorption, nickel dithiolenes have been suggested to be candidates for NIR sensitizers in DSSCs [2,10]. Except for the suitable absorption wavelength and the excited energy, the efficient sensitizers for DSSCs should have a large LHE. LHE prominently affects the IPCE. To our knowledge, the LHE of nickel dithiolenes has not been reported yet. The LHE values of Ni[S_2_C_2_(CN)_2_]_2_, Ni[S_2_C_2_(NO_2_)_2_]_2_, Ni[S_2_C_2_(CH=CH_2_)_2_]_2_, and Ni[S_2_C_2_(C≡CH)_2_]_2_ were calculated to be in the range of 0.3530–0.4903, which were higher than those of the recently reported noble metal ruthenium-based dyes in DSSCs [35]. The LHE values of Ni[S_2_C_2_(OH)_2_]_2_ and Ni[S_2_C_2_(NH_2_)_2_]_2_ were too small to be as efficient DSSCs although they have the maximum absorption wavelength. It is reported that the main problem of nickel dithiolenes to be used as the material for DSSCc lies in that the lifetime of excited states is not long enough and the efficiency is still low. Herein, the charge injection efficiency into the semiconductor layer should be very high as the excited state is not long lived [19]. Nickel dithiolenes with conjugated groups have both high EA and high LHE, therefore, both the electron injection efficiency and the light harvesting efficiency are high. Therefore, they are promising candidates as materials for the NIR sensitizer of DSSCs.

**Figure 2:**
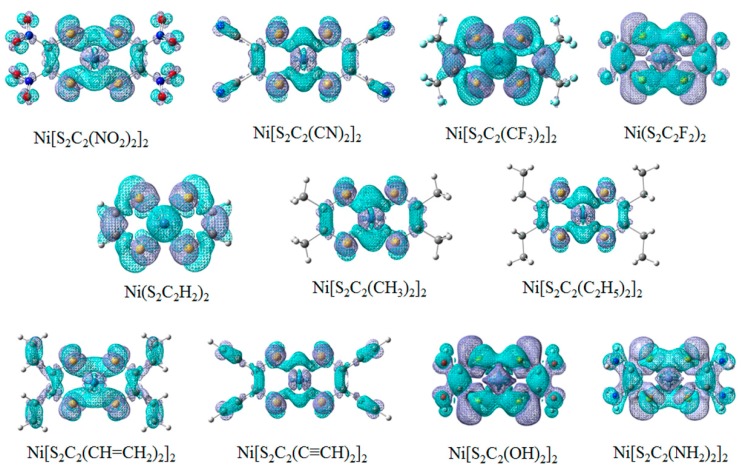
The electron density of Ni(S_2_C_2_R_2_)_2_ (R =–NO_2_, –CN, –CF_3_, –F, –H, –CH_3_, –C_2_H_5_, –CH=CH_2_, –C≡CH, –OH, –NH_2_) in the ground state and the first excited state. The electron density is in the unit of e/Å^3^. S is represented as yellow ball; Ni is represented as light blue ball; N is represented as dark blue ball; C is represented as gray ball; H is represented as white ball; O is represented as red ball.

We studied the contribution of electron transition from the ground state to the first excited state by comparing the electron density of the ground state to that of the first excited state. In this process, electron flew from the blue part (negative value) to the purple part (positive value). The electronic transition from the ground state to the first excited state is attributed to the *π*-π* transition. The LUMO and HOMO were *π*-antibonding and *π*-bonding orbitals mainly from C and S atoms in ligands, and a little from Ni atom. Most substituents contributed to the electron flow from the ground state to the excited state (Figure 2). The unsaturated groups or groups with the p orbital conjugated with dithiolenes such as –NO_2_, –CN, –CH=CH_2_, –C≡CH, –OH, –NH_2_. The substituents contributed greatly to the electron flow from the ground state to the excited state, and thus significantly affected the electron transition from the ground state to the first excited state.

### 3.5. The Electron Injection and Transportation Controlled by the Substituents

**Figure 3:**
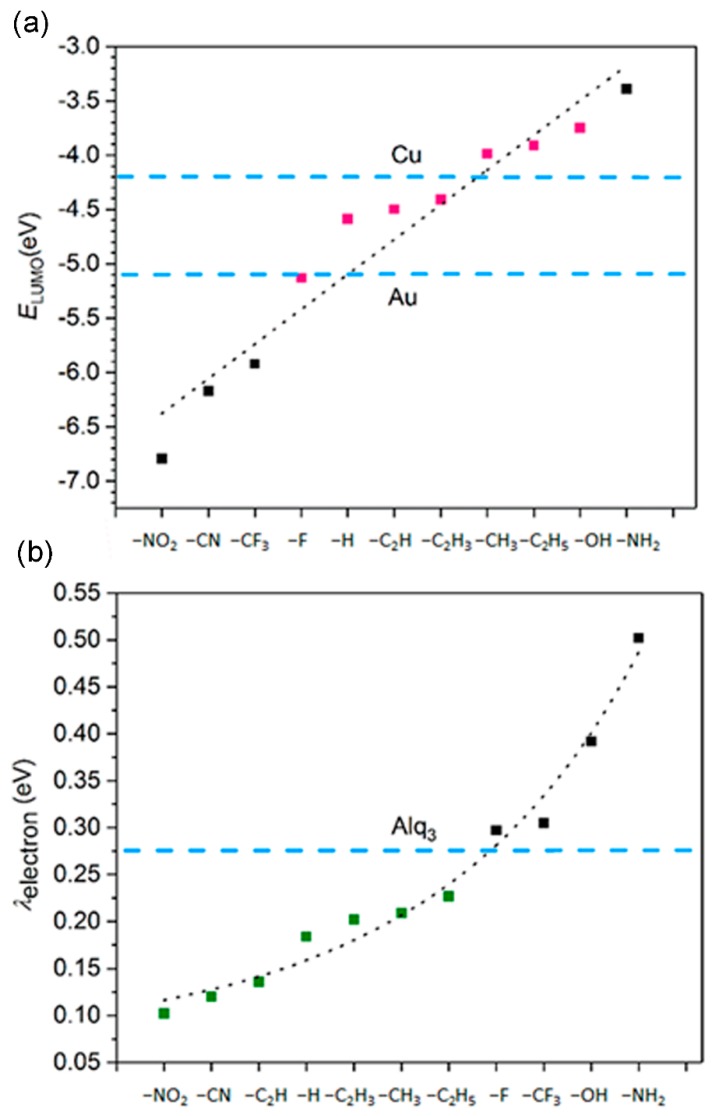
(**a**) Linear regression of the LUMO energies of substituted nickel dithiolenes with the Hammett’s constant of substituents. The blue dash line represents the work function of Au or Cu electrode. (**b**) The electron reorganization energies of substituted nickel dithiolenes, and the bluedash line represents the electron reorganization energy of the commonly used electron transportation layer material Alq3, which is the aluminum salt of Tris(8-hydroxyquinolin). Alq3 can be used as both organic electroluminescent materials and electron transportation layer materials.

Based on the calculation results, we brought to light the correlation between the substituents of nickel dithiolenes and their efficient electron injection and transportation ability. We found that the LUMO energy of nickel dithiolenes increases with the Hammett’s constant [36] of their substituents (Figure 3a). Thus, we are able to modify the energy level of LUMO by choosing the substituents with proper electron-donating ability. For example, in the present study the efficient electron injection requires that the LUMO energies match the work function of electrode. So we used nickel dithiolenes with weak electron-donating groups (such as –CH_3_, –C_2_H_5_, –C_2_H_3_, –C_2_H) to match the work function of Cu electrode.

The efficient electron transportation is characterized by the low reorganization energy. We find that the electron reorganization energies correlate with the conjugation effect between the dithiolene rings and the substituents. The unsaturated groups such as –NO_2_, –CN, and –C≡CH are likely to form conjugation with dithiolene rings, so that they have low reorganization energies than the others (Figure 3b).

Nickel dithiolenes with suitable LUMO energy level and low reorganization energy are ideal for electron injection and transporting layer material for n-type semiconductor and the NIR sensitizer of DSSCs. Based on the correlation between the substituents of nickel dithiolenes and their efficient electron injection and transportation ability, we report in the present study that nickel dithiolenes could be used as such ideal materials.

## 4. Conclusions

The electronic and optical properties of nickel dithiolene and its derivatives were investigated based on DFT and TD-DFT. According to our research, the IP and EA values of these molecules increased with the electron-withdrawing ability of the substituents. The EA(a) values of these complexes were very high. Thus, they can be used as the electron injection layer material. The reorganization energies of these complexes with unsaturated groups were smaller than the commonly used electron transfer material (tris-8-hydroxyquinolate-aluminum). Herein they are potential electron transportation materials for n-type organic semiconductor. The maximum absorption wavelengths of nickel dithiolenes were in the visible or NIR region. Both the electron injection efficiency and the light harvesting efficiency of the nickel dithiolenes were high. Therefore, they are promising candidates as the materials for NIR sensitizer of DSSCs. We calculated the electron density of the ground state and the first excited state and found that the first excited state of these complexes was assigned to π-π* transition mainly from HOMO to LUMO. In sum, all these data provide a theoretical basis for applying nickel dithiolenes as the materials for n-type semiconductors and NIR sensitizers.

## Figures and Tables

**Table 1:** The energies, the energy gaps between highest occupied molecular orbital (HOMO) and lowest unoccupied molecular orbital (LUMO), the *λ*_hole_ and *λ*_electron_ for Ni(S_2_C_2_R_2_)_2_. (R=–NO_2_, –CN, –CF_3_, –F, –H, –CH_3_, –C_2_H_5_, –CH=CH_2_, –C≡CH, –OH, –NH_2_).

Substituent	*E*_HOMO_/eV	*E*_LUMO_/eV	Δ*E*_H−L_/eV
–NO_2_	−8.307	−6.793	1.514
–CN	−7.796	−6.173	1.623
–CF_3_	−7.717	−5.922	1.795
–F	−6.761	−5.130	1.631
–H	−6.158	−4.410	1.748
–CH_3_	−5.581	−3.910	1.671
–C_2_H_5_	−5.442	−3.749	1.693
–CH=CH_2_	−5.933	−4.498	1.435
–C≡CH	−6.108	−4.590	1.518
–OH	−5.263	−3.988	1.275
–NH_2_	−4.593	−3.389	1.204

**Table 2:** The ionization potentials, electron affinities and reorganization energies of Ni(S_2_C_2_R_2_)_2_. (R = –NO_2_, –CN, –CF_3_, –F, –H, –CH_3_, –C_2_H_5_, –CH=CH_2_, –C≡CH, –OH, –NH_2_).

Substituent	IP(v)/eV	IP(a)/eV	EA(v)/eV	EA(a)/eV	*λ*_hole_/eV	*λ*_electron_/eV
–NO_2_	9.44	9.27	4.87	4.97	0.181	0.102
–CN	9.21	9.11	4.75	4.81	0.211	0.120
–CF_3_	9.08	8.87	4.17	4.33	0.418	0.305
–F	8.23	8.03	3.37	3.52	0.391	0.297
–H	7.83	7.71	2.76	2.85	0.245	0.184
–CH_3_	7.06	6.93	2.41	2.51	0.248	0.202
–C_2_H_5_	6.82	6.69	2.31	2.43	0.255	0.227
–CH=CH_2_	6.92	6.83	2.89	3.01	0.179	0.209
–C≡CH	7.37	7.28	3.28	3.35	0.177	0.136
–OH	6.81	6.60	2.42	2.61	0.430	0.392
–NH_2_	6.05	5.77	1.92	2.13	0.574	0.502

**Table 3:** The maximum absorption wavelength of Ni(S_2_C_2_R_2_)_2_. (R=–NO_2_, –CN, –CF_3_, –F, –H, –CH_3_, –C_2_H_5_, –CH=CH_2_, –C≡CH, –OH, –NH_2_).

Substituent	*λ*/nm	Excitation Energy/eV	Oscillator Strength	LHE
–NO_2_	751.23	1.6504	0.1891	0.3530
–CN	774.91	1.6000	0.2231	0.4017
–CF_3_	688.66	1.8004	0.1587	0.3061
–F	740.17	1.6751	0.1447	0.2834
–H	670.76	1.8484	0.1372	0.2709
–CH_3_	707.58	1.7522	0.2006	0.3699
–C_2_H_5_	726.68	1.7062	0.2151	0.3906
–CH=CH_2_	859.00	1.4434	0.2644	0.4560
–C≡CH	823.66	1.5053	0.2927	0.4903
–OH	822.66	1.5071	0.1868	0.3496
–NH_2_	1022.86	1.2121	0.0706	0.1500

## Supplementary Materials

The following are available online at http://www.mdpi.com/1996-1944/11/11/2192/s1, Figure S1: Chemical structure of substituted nickel dithiolenes (R=–CN, –H, –CH_3_); Table S1: The bond lengths (Å) and bond angles (°) of Ni(S_2_C_2_R_2_)_2_. (R = –CN, –H, –CH_3_) using different functionals (the available experimental data is in italics), Table S2: The maximum absorption wavelength (cm^−1^) of Ni(S_2_C_2_H_2_)_2_ in hexane (the available experimental data is in italics), Table S3: The frontier molecular orbitals of Ni(S_2_C_2_R_2_)_2_ optimized by Gaussian 09 software. (R = –NO_2_, –CN, –CF_3_, –F, –H, –CH_3_, –C_2_–H_5_, –CH=CH_2_, –C≡CH, –OH, –NH_2_).
