# Supplementary material for: Regulating the Optoelectronic Properties of Nickel Dithiolene by the Substituents: A Theoretical Study
Source: Materials (Basel). 2018 Nov 6;11(11):2192. doi: 10.3390/ma11112192 (PMC6265744; doi:10.3390/ma11112192)
Supplement: Supplementary file 1 [file materials-11-02192-s001.pdf]

materials

Supplementary

Regulating the Optoelectronic Properties of Nickel Dithiolene by the Substituents: A Theoretical Study

Lili Sun, Siwei Shu, Yi Zhou, Sen Hou, Yan Liu and Zhuofeng Ke

Verification of the Computational Method

In order to choose reasonable basis functional for this work, we compared some simulation results with the available experimental data in the literature. We chose four kinds of basis functionals (M06, M06L, B3LYP, PBEPBE) to perform geometry optimization for $Ni(S_2C_2H_2)_2$, $Ni[S_2C_2(CH_3)_2]_2$ and $Ni[S_2C_2(CN)_2]_2$ and performed TD-DFT calculation for $Ni(S_2C_2H_2)_2$. We chose the same basis set with the literature for Ni [1], in which the LANL2DZ basis set was adopted for Ni, the two outermost p functions were replaced by a (41) split of optimized p polarization function of Ni and an f function was added to Ni as it is more helpful for predicting excited states accurately. The 6-311 + G* was adopted for other atoms except Ni.

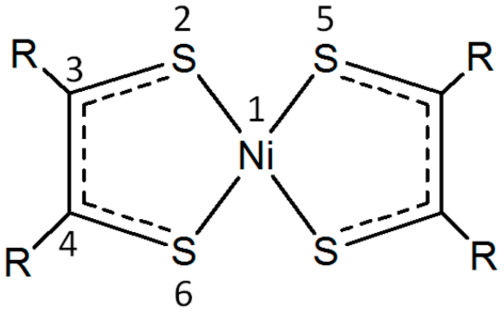

Figure. S1 Chemical structure of substituted nickel dithiolenes. (R=–CN, –H, $-CH_3$).

Table S1 The bond lengths (Å) and bond angles (°) of $Ni(S_2C_2R_2)_2$. (R = –CN, –H, $-CH_3$) using different functionals (the available experimental data is in italics).

Substituent	Functional	C3–S2	S2–Ni1	C3–C4	∠S2Ni1S6	∠S2Ni1S5
R=–H	M06	1.705	2.178	1.373	92.08	87.92
R=–H	M06L	1.704	2.178	1.373	92.08	87.91
R=–H	PBEPBE	1.705	2.178	1.373	92.08	87.92
R=–H	B3LYP	1.705	2.178	1.373	92.08	87.91
Experimental data [2]		*1.71*	*2.15*		*92*	*88*
R=$-CH_3$	M06	1.706	2.161	1.385	91.73	88.27
R=$-CH_3$	M06L	1.707	2.178	1.388	90.92	89.08
R=$-CH_3$	PBEPBE	1.721	2.174	1.395	91.01	88.98
R=$-CH_3$	B3LYP	1.715	2.169	1.389	90.95	89.05
Experimental data [3]		*1.714*	*2.128*	*1.365*	*91.35*	
R=–CN	M06	1.701	2.164	1.393	92.96	87.04
R=–CN	M06L	1.703	2.180	1.401	92.11	87.89
R=–CN	PBEPBE	1.718	2.178	1.409	92.25	87.75

R=–CN	B3LYP	1.711	2.171	1.400	92.12	87.88
Experimental data [2]		*1.72*	*2.15*	*1.37*	*92.5*	*87.5*

Table S2 The maximum absorption wavelength (cm^{-1}) of $Ni(S_2C_2H_2)_2$ in hexane (the available experimental data is in italics).

	Functional	λ/nm
R=–H	M06	748.59
R=–H	M06L	856.37
R=–H	PBEPBE	841.78
R=–H	B3LYP	714.15
Experimental data [4]		*719*

The Contour of Frontier Molecular Orbitals

Table S3 The frontier molecular orbitals of $Ni(S_2C_2R_2)_2$ optimized by Gaussian 09 software. (R=–NO_2, –CN, –CF_3, –F, –H, –CH_3, –C_2–H_5, –CH=CH_2, –C≡CH, –OH, –NH_2).

Substituent	LUMO	HOMO
–NO_2	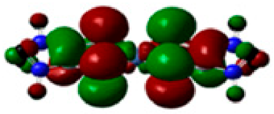	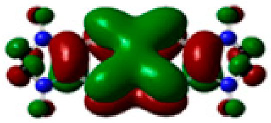
–CN	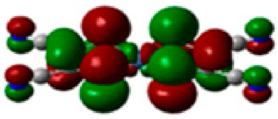	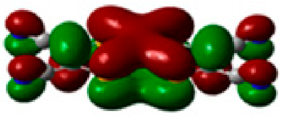
–CF_3	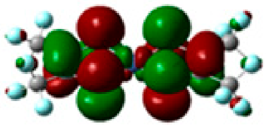	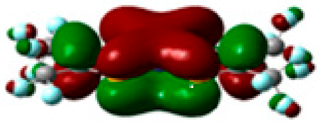
–F	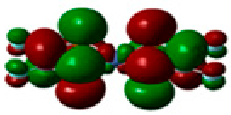	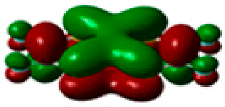
H	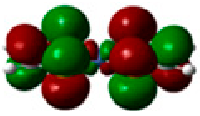	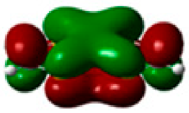
–CH_3	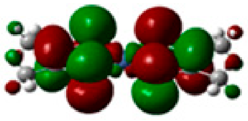	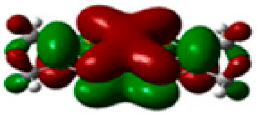
–C_2H_5	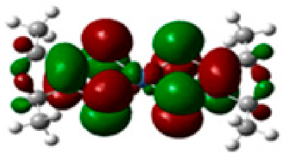	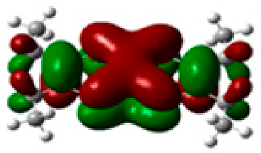

$–CH=CH_2$	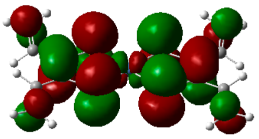	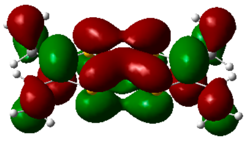
–C≡CH	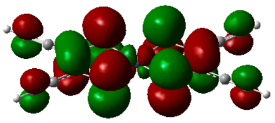	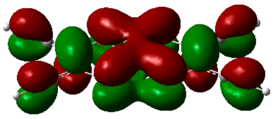
–OH	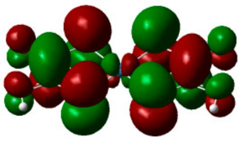	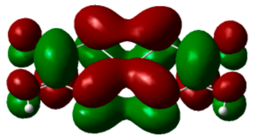
$–NH_2$	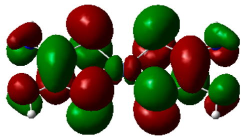	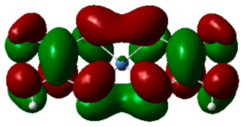

References

1. Fan, Y.B.; Hall, M.B. How electron flow controls the thermochemistry of the addition of olefins to nickel dithiolenes: Predictions by density functional theory. *J. Am. Chem. Soc.* **2002**, *124*, 12076–12077.
2. Eisenberg, R. *Progress in Inorganic Chemistry*; John Wiley and Sons Ltd: New York, NY, USA, 1970; Vol. 12.
3. Lim, B.S.; Fomitchev, D.V.; Holm, R.H. Nickel dithiolenes revisited: Structures and electron distribution from density functional theory for the three-member electron-transfer series $[Ni(S_2C_2Me_2)_2]^{0, 1-, 2-}$. *Inorg. Chem.* **2001**, *40*, 4257–4262.
4. Lauterbach, C.; Fabian, J. Density functional derived structures and molecular properties of nickel dithiolenes and related complexes. *Eur. J. Inorg. Chem.* **1999**, *11*, 1995–2004.

© 2018 by the authors. Licensee MDPI, Basel, Switzerland. This article is an open access article distributed under the terms and conditions of the Creative Commons Attribution (CC BY) license (http://creativecommons.org/licenses/by/4.0/).
